# Species Identification of Marine Fishes in China with DNA Barcoding

**DOI:** 10.1155/2011/978253

**Published:** 2011-05-11

**Authors:** Junbin Zhang

**Affiliations:** College of Fisheries and Life Science, Shanghai Ocean University, Shanghai 201306, China

## Abstract

DNA barcoding is a molecular method that uses a short standardized DNA sequence as a species identification tool. In this study, the standard 652 base-pair region of the mitochondrial cytochrome oxidase subunit I gene (COI) was sequenced in marine fish specimens captured in China. The average genetic distance was 50-fold higher between species than within species, as Kimura two parameter (K2P) genetic distances averaged 15.742% among congeners and only 0.319% for intraspecific individuals. There are no overlaps of pairwise genetic variations between conspecific and interspecific comparisons apart from the genera *Pampus* in which the introgressive hybridization was detected. High efficiency of species identification was demonstrated in the present study by DNA barcoding. Due to the incidence of cryptic species, an assumed threshold is suggested to expedite discovering of new species and biodiversity, especially involving biotas of few studies.

## 1. Introduction

Fishes are important animal protein sources for human beings, and they are frequently used in complementary and alternative medicine/traditional medicine (CAM/TM). The delimitation and recognition of fish species is not only of interest for taxonomy and systematics, but also a requirement in management of fisheries, authentication of food products, and identification of CAM/TM materials [1–3].

 Due to the complexity and limitations of morphological characters used in traditional taxonomy, several PCR-based methods of genotype analysis have been developed for the identification of fish species, particularly for eggs, larvae, and commercial products. Sequence analysis of species-specific DNA fragments (often mitochondrial or ribosomal genes) and multiplex PCR of species-conserved DNA fragments are efficient for fish species identification [4–10]. However, these molecular methods are limited to particular known species and are not easily applicable to a wide range of taxa. Therefore, Hebert et al. advocated using a standard DNA sequence that is DNA barcoding to identify species and uncover biological diversity [11, 12]. For many animal taxa, sequence divergences within the 5′ region of the mitochondrial cytochrome oxidase subunit I (COI) gene were much greater between species than within them, and this in turn suggests that the approach is widely applicable across phylogenetically distant animal groups [12, 13]. To date, some published papers explicitly address that COI barcodes effectively discriminate different species for a variety of organisms [14–23]. However, several scientists express concerns that species identification based on variations of single mitochondrial gene fragment may remain incorrect or ambiguous assignments, particularly in cases of possible mitochondrial polyphyly or paraphyly [24, 25]. In the current study, we test the efficacy of DNA barcoding in marine fishes of China. The sea area of China is part of the Indo-West Pacific Ocean, which is regarded as the center of the world's marine biodiversity [26]. Highly species-rich biotas are particularly attractive to test the reliability and efficiency of DNA barcoding.

## 2. Material and Methods

The majority of fish specimens were captured with the drawl net at 20 localities along the coast of China (collection information available at http://www.barcodinglife.org/). A total of 329 specimens from one hundred species of fish were collected. Vouchers were deposited in the South China Sea Institute of Oceanography, Chinese Academy of Sciences, and all specimens were preserved in 70% ethanol. Tissue samples were dissected from the dorsal muscle, and genomic DNA was extracted according to the standard Barcode of Life protocol [27]. Firstly, fragments of the 5′ region of the mitochondrial COI gene were PCR-amplified using C_FishF1t1/ C_FishR1t1 primer cocktails [28]. The cocktail C_FishF1t1 contained two primers (FishF2_t1/VF2_t1), and C_FishR1t1 also contained two primers (FishR2_t1/ FR1d_t1). All PCR primers were tailed with M13 sequences to facilitate sequencing of products. The nucleotide sequences of the primers were

FishF2_t1: *5′-TGTAAAACGACGGCCAGTCGACTAATCATAAAGATATCGGCAC-3′.
VF2_t1: *5′-TGTAAAACGACGGCCAGTCAACCAACCACAAAGACATTGGCAC-3′.
FishR2_t1:**5′-CAGGAAACAGCTATGACACTTCAGGGTGACCGAAGAATCAGAA-3′.
FR1d_t1: **5′-CAGGAAACAGCTATGACACCTCAGGGTGTCCGAARAAYCARAA-3′.
*The M13F primer sequence is underlined;**the M13R primer sequence is underlined.

PCR reactions were carried out in 96-well plates using Mastercycler Eppendorf gradient thermal cyclers (Brinkmann Instruments, Inc.). The reaction mixture of 825 *μ*l water, 125 *μ*l 10× buffer, 62.5 *μ*l MgCl_2_ (25 mM), 6.25 *μ*l dNTP (10 mM), 6.25 *μ*l each primer (0.01 mM), and 6.25 *μ*l Taq DNA polymerase (5 U/*μ*l) was prepared for 96 wells of each plate, in which each well contained 10.5 *μ*l mixture and 2 *μ*l genomic DNA. Thermocycling comprised an initial step of 2 min at 95°C and 35 cycles of 30 sec at 94°C, 40 sec at 52°C, and 1 min at 72°C, with a final extension at 72°C for 10 min. Amplicons were visualized on 2% agarose E-Gel 96-well system (Invitrogen). PCR products were amplified again with the primers M13F (5′-TGTAAAACGACGGCCAGT-3′) and M13R (5′-CAGGAAACAGCTATGAC-3′), respectively, using the BigDye Terminator v.3.1 Cycle Sequencing Kit (Applied Biosystems, Inc.). Thermocycling conditions were as follows: an initial step of 2 min at 96°C and 35 cycles of 30 sec at 96°C, 15 sec at 55°C, and 4 min at 60°C. Sequencing was performed on an ABI 3730 capillary sequencer according to manufacturer's instructions. 

For specimens that failed to yield sequences using the primer combinations above, a second round of PCR using the alternative C_VF1LFt1/ C_VR1LRt1 primer combination was carried out. C_VF1LFt1 consisted of four primers (VF1_t1/VF1d_t1/LepF1_t1/VFli_t1), and C_VR1LRt1 also comprised four primers (VR1_t1/VR1d_t1/LepR1_t1/VRli_t1) [28].

  VF1_t1: *5′-TGTAAAACGACGGCCAGTTCTCAACCAACCACAAAGACATTGG-3′.
  VF1d_t1: *5′-TGTAAAACGACGGCCAGTTCTCAACCAACCACAARGAYATYGG-3′.
LepF1_t1: *5′-TGTAAAACGACGGCCAGTATTCAACCAATCATAAAGATATTGG-3′.
VFli_t1: *5′-TGTAAAACGACGGCCAGTTCTCAACCAACCAIAAIGAIATIGG-3′.
VR1_t1: **5^'^-CAGGAAACAGCTATGACTAGACTTCTGGGTGGCCRAARAAYCA-3′.
VR1d_t1: **5′-CAGGAAACAGCTATGACTAGACTTCTGGGTGGCCAAAGAATCA-3′.
LepR1_t1: **5′-CAGGAAACAGCTATGACTAAACTTCTGGATGTCCAAAAAATCA-3′.
VRli_t1: **5′-CAGGAAACAGCTATGACTAGACTTCTGGGTGICCIAAIAAICA-3′.
*The M13F primer sequence is underlined;**the M13R primer sequence is underlined.

The thermocycling protocol used was 1 min at 95°C and 35 cycles of 30 sec at 94°C, 40 sec at 50°C, and 1 min at 72°C, with a final extension at 72°C for 10 min. Sequecing PCR and sequencing followed above procedure.

DNA sequences were aligned with SEQSCAPE v.2.5 software (Applied Biosystems, Inc.). Sequence divergences were calculated using the Kimura two parameter (K2P) distance model [29], and unrooted NJ trees based on K2P distances were created in MEGA software [30]. In the chosen taxonomic group, phylogenetic analysis was carried out in PAUP 4.010b using the maximum parsimony (MP) method, with 1,000 replications of the full heuristic search.

The following categories of K2P distances were calculated: intraspecific distances (S), interspecies within the congener (G), and interspecies from different genus but within intrafamily (F). These values were plotted using the boxplot representation of R. Boxplots [31] in SPSS 11.5 software (SPSS Inc., Chicago, IL, USA). Only for families containing 2 or more genera, separate boxplot was constructed for the sake of comparisons among taxonomic categories. Boxplots describe median (central bar), interquartile range (IQR: between upper (Q3) and low (Q1) quartile), values lying within 1.5× IQR beneath Q1 or 1.5× above Q3 (“whiskers”), and extreme values (outliers). Mann-Whitney tests were performed between S, G, and F distributions to estimate the overlap among taxonomic ranks.

## 3. Results

A total of 329 specimens were analyzed, from which 321 sequences (all >500 bp) belonging to 121 species (another species was identified to the genus level) were ultimately obtained (GenBank accession numbers: EF607296-EF607616). These species cover the majority of fishes living in the coastline of the South China Sea. All sequences were aligned with a consensus length of 652 bp, and no insertions, deletions, or stop codons were observed in any sequence. However, multiple haplotypes were detected for some species.

Except for *Acentrogobius caninus, Scomber japonicus, Terapon jarbua, Upeneus sulphureus, Elops hawaiensis, Gymnothorax pseudothyrsoideus, Dendrophysa russelii, and Pennahia anea* (which reached the maximum value of 2.02%), intraspecific genetic distances were generally below 1%, and some decreased to zero (between some intraspecific individuals of *Thryssa setirostris, Parapercis ommatura, Scatophagus argus,* etc.). 

**Figure 1:**
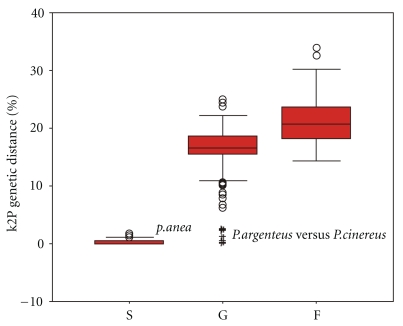
Box plots of K2P distances. IQR: interval into which the “central” 50% of the data fall. Black bar in the box indicates the median. Circle: “mild outlier” and asterisks: “extreme outliers”. Extreme outliers are discussed in the text.

**Figure 2:**
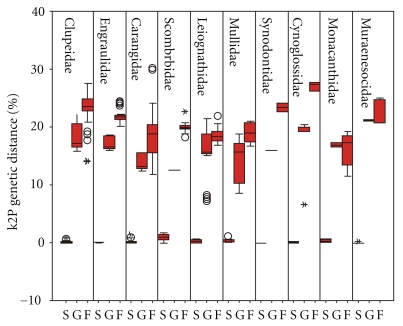
Boxplot distributions of S, G, and F. Intra-species (S), interspecies among congeneric species (G), and intergenera but intrafamily (F) K2P distances for different families.

The mean intraspecies K2P (Kimura two-parameter) distance was 0.319%; the distance increased sharply to 15.742% among individuals of congeneric species. Overall, the average genetic distance among congeneric species is nearly 50-fold higher than that among individuals within species. For the higher taxonomic ranks (family, order, and class), mean pairwise genetic distances increased gradually and reached 20.199%, 24.656%, and 25.225%, respectively (Table 1). Standard errors for K2P genetic distances were small, and values of the mean and median were close within different taxonomic ranks (Table 1). This indicates fluctuations of K2P genetic distances tend to be convergent (Figures 1 and 2).

**Figure 3:**
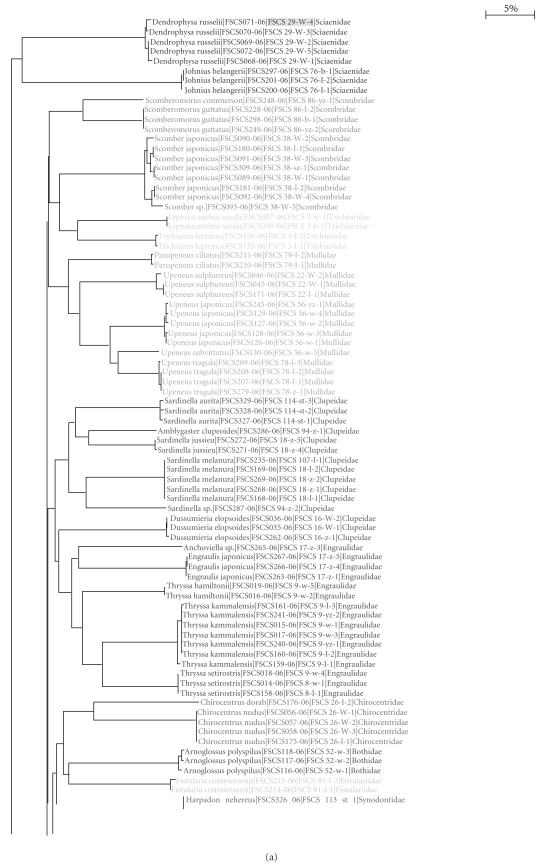

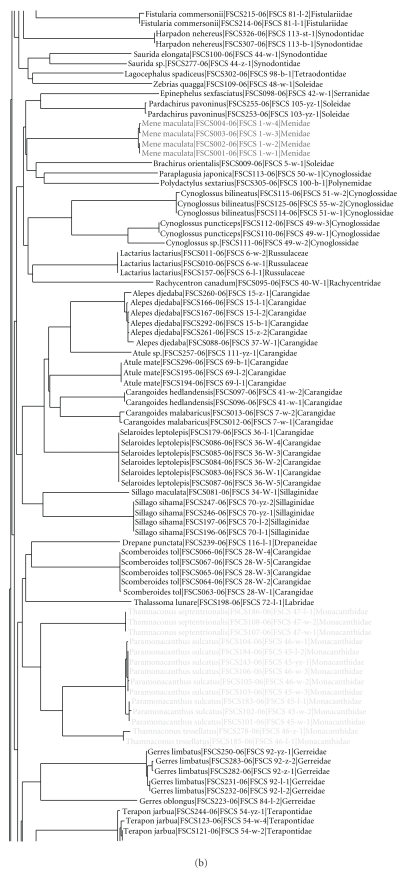

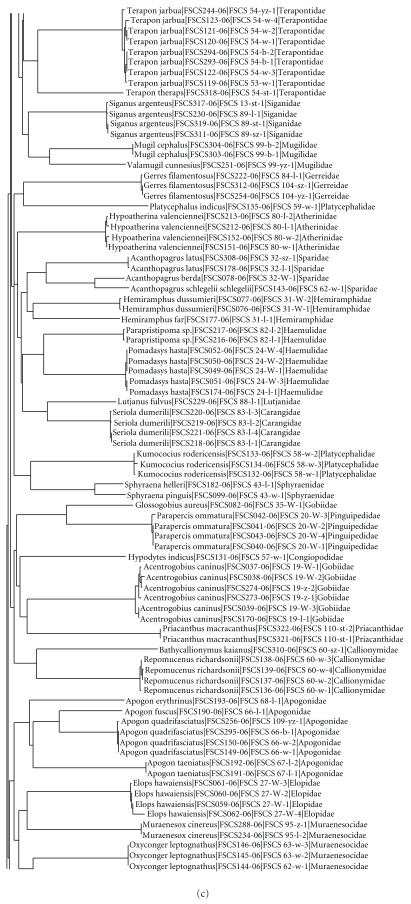

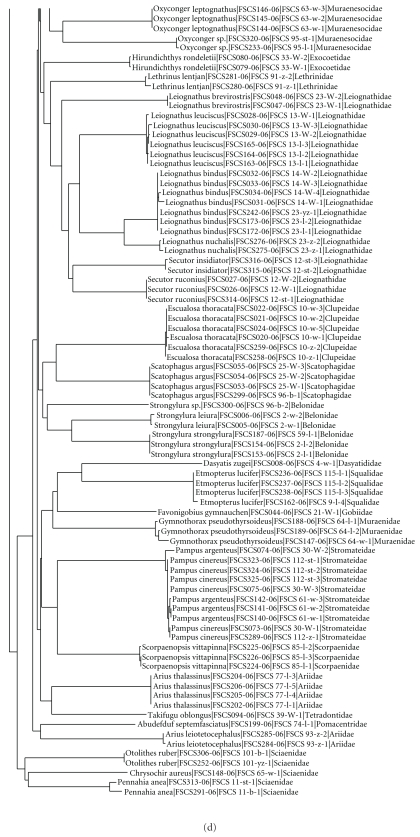
Neighbor-joining (NJ) tree of COI sequences. Scale: 5% K2P distance. The first numbers following species names are the process IDs, and the latter are the sample IDs.

In the unrooted NJ (neighbour-joining) tree (Figure 3), three specimens of *Pampus argentenus *were grouped together and contained within the cluster of *Pampus cinereus*. These *Pampus argentenus* specimens were collected in the same site off the west coast of the South China Sea, and were difficult to identify because of their complex morphological characteristics (available at http://www.barcodinglife.org/). They possessed combined characteristics of *Pampus cinereus* and *Pampus argentenus*: the asymmetrical tail of *Pampus cinereus* and silver color of *Pampus argentenus*. If the suspicious congeneric K2P distances in the genera *Pampus *are excluded (the extreme outliers in Figure 1), the pairwise genetic divergences among congeneric species are above 10%. There are no overlaps between intraspecific and congeneric K2P distances within the same family (Figure 3).

At the species level, all COI sequences clustered in monophyletic species units. At the family level, there were paraphyletic clusters for three families (Carangidae, Gobiidae, and Ariidae) (Figure 3), though over 98% of specimens fell into the expected division of families. Intrafamily K2P distances (F) were generally higher than congeneric (G) distances, which were definitely higher than intraspecific (S) distances (Table 1, all Mann-Whitney tests were highly significant, P value <10−6). However, overlaps between F and G distances were observed in Clupeidae, Carangidae, Mullidae, and Muraenesocidae.

## 4. Discussion

In morphological taxonomy, characters are delimited usually without any explicit criteria for character selection or coding, and morphological data sets have the potential to be quite arbitrary. For example, morphologists do not generally report their criteria for including or excluding characters, and when criteria are given, they vary considerably among studies [32]. Thus, it is not surprising that there are so many synonyms for organisms [33], and an objective, rigorous species delimitation according to explicit criteria is therefore necessary for many taxonomic studies [34]. While DNA barcoding provides taxonomic identification for a specimen, the accuracy of such an assignment depends on whether species are monophyletic with respect to sequence variations of the COI gene. That is, individuals of a given species are more closely related to all other conspecifics than to any member of other species. Except for the hybridized specimens in the genus Pampus, there are no overlaps between genetic variations of S and G (Figure 1).

The factors responsible for deviations from taxonomic monophyly may be varied and complex [35]; one potential cause of species-level polyphyly is the occasional mating between distinct species, resulting in hybrid offspring carrying a mixture of genes from both parent species. Furthermore, mitochondrial genes are generally subjected to introgression more frequently than nuclear ones, and introgression also leads to phylogenetic paraphyly [35–38], like the hybridization between *Pampus argentenus* and *Pampus cinereus* in this study. In such cases, combinations of morphological and genotypic data are needed for species assignment of hybrids.

Biological mechanisms, water dynamics, or historical events may cause deep genetic structuring of populations in marine species [26, 39]. Many explanations for genetic population structuring on local and regional scales involve behaviors such as the adoption of pelagic early life stages and movement over broad geographic ranges, and these factors are theoretically associated with gene flow [40–42]. For many marine fishes, there is a lack of phylogeographic structure among populations [43, 44]; in this study, for individuals from long distance localities, some intraspecific genetic variations reduced to zero within families Carangidae, Sciaenidae, and Mullidae. However, some pairwise K2P distances exceeded 1.00% within the coastal species such as *Acentrogobius caninus, Scomber japonicus, Terapon jarbua, Upeneus sulphureus, Elops hawaiensis, Gymnothorax pseudothyrsoideus, and Dendrophysa russelii*. It implied that biological mechanisms were responsible for the fluctuation of intraspecific genetic divergences in marine fishes. 

The neighbor-joining method was originally employed in this study for species identification, but some phylogenetic information was also revealed by the dendrogram, and over 98% of specimens were allocated into different families without polyphyly/paraphyly in the NJ tree (Figure 3). However, DNA barcoding is independent of the way the taxonomy has been built, and it cannot be regarded as the “taxonomic” tag [45]. DNA barcoding is no substitute for taxonomy Ebach and Holdrege [46], and a great deal of work is needed to bring about the reconciliation between traditional and molecular taxonomy. It is unfeasible to build the phylogeny of fishes only based on mitochondrial DNA fragments. Polyphyly/paraphyly in the NJ tree probably results from “bad taxonomy” when named species fail to identify the genetic limits of separate evolutionary entities, particularly for perplexing taxa involving cryptic species [47]. If we cannot set a threshold of the genetic variation in species delimitation, we find ourselves sunk in the dilemma facing new or cryptic species. On the one hand, the morphological taxonomy cannot give a definite identification. On the other hand, we cannot claim that it may be a new species based on molecular analysis without the species delimitation. An assumed threshold is helpful to expedite discovery of new species and biodiversity, especially in dealing with little-studied biotas, although a single, uniform threshold for species delimitation seems arbitrary because rates of molecular evolution vary widely within and among lineages [24, 25, 48].

## Figures and Tables

**Table 1:** Genetic divergences (percentage, K2P distance) within various taxonomic levels. Data are based on 321 sequences (>500 bp) from 122 species.

Comparisons within	Taxa	Number of comparisons	Mean	Median	Minimum	Maximum	s.e.^#^
Species	121	453	0.319	0.150	0	2.021*	0.018
Genus	85	397	15.742	16.490	0.154**	25.189	0.292
Family	55	848	20.199	19.850	11.532	34.333	0.134
Order	15	17881	24.656	—	12.923	39.627	0.024
Class	2	29262	25.225	—	15.730	40.800	0.016

* Pennahia anea; ** Pampus argenteus versus Pampus cinereus.

^#^Standard error.
